# Research progress in vaccination during pregnancy

**DOI:** 10.3389/fimmu.2025.1687362

**Published:** 2025-10-29

**Authors:** Jiao Zhang, Zhimin Li, Shiran Li, Pengfei Li, Jingxian Xie, Yong Yang

**Affiliations:** ^1^ Department of Pharmacy, Personalized Drug Research and Therapy Key Laboratory of Sichuan Province, Sichuan Provincial People’s Hospital, School of Medicine, University of Electronic Science and Technology of China, Chengdu, China; ^2^ Department of Pharmacy, Chongqing Red Cross Hospital (People’s Hospital of Jiangbei District), Chongqing, China; ^3^ Department of Pharmacy, Bishan Hospital of Chongqing Medical University (Bishan Hospital of Chongqing), Chongqing, China; ^4^ School of Pharmacy, Southwest Medical University, Luzhou, China

**Keywords:** pregnancy, vaccination, maternal immunization, vaccine safety, vaccine efficacy

## Abstract

Pregnant women and infants are more vulnerable to infectious diseases than the general population. Vaccination during pregnancy can protect not only mothers and fetuses from diseases but also safeguard infants through maternal antibodies transferred *via* the placenta. It stands as one of the most effective strategies to reduce the incidence and mortality of infectious diseases among pregnant women and infants. Globally, pregnancy vaccination strategies are increasingly diversified. This review discusses the effectiveness and safety of various vaccines for preventing infectious diseases during pregnancy, as well as vaccination recommendations for pregnant women across different countries.

## Introduction

**Figure 1:**
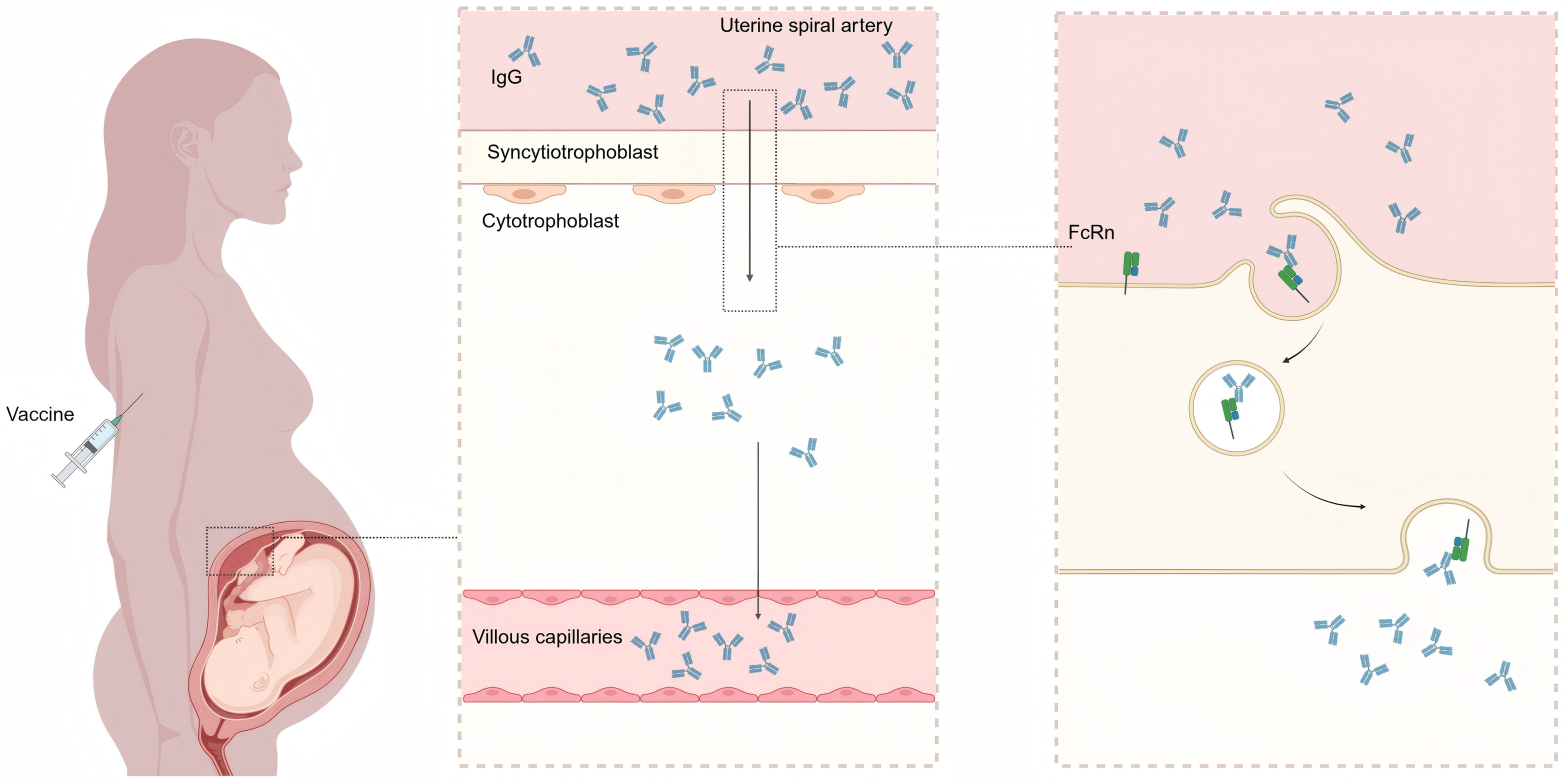
Mechanism of transplacental IgG transfer following maternal vaccination. Maternal IgG antibodies (produced postvaccination) bind to the fragment crystallizable neonatal receptor (FcRn) on placental trophoblast cells, undergo transcytosis across the syncytiotrophoblast layer, and are subsequently released into the fetal circulation. This figure was created with BioRender (https://biorender.com/).

The immature immune system of fetuses and neonates renders them highly susceptible to infectious pathogens. Key factors contributing to this vulnerability include underdeveloped adaptive immunity, reduced antigen-presenting capacity, lack of immunological memory, and incomplete formation of mucosal barriers. These deficiencies result in a diminished capacity to combat microbial infections, often leading to more severe clinical manifestations and prolonged disease courses compared to adults. Maternal immunization represents a critical public health intervention to protect both mothers and their offspring. By eliciting pathogen-specific antibodies, vaccination during pregnancy facilitates transplacental transfer of immunoglobulin G (IgG), conferring passive immunity to fetuses and neonates during their period of highest susceptibility (**Figure 1**). Some vaccines have been proven safe and effective and are widely used in pregnant women, such as the tetanus vaccine, pertussis vaccine, and influenza vaccine. Other vaccines have varying recommendations for pregnant women across different countries or regions, while the effectiveness and safety of some vaccines in pregnant women remain controversial or under research. To provide a clear overview of the vaccines discussed in this review, **Table 1** summarizes types, components, and key information for each vaccine—including their target pathogens, efficacy, safety profiles, and major recommendations for use during pregnancy.

**Table 1:** Summary of key vaccines for infectious disease prevention during pregnancy.

Vaccine type	Type of vaccine	Components of vaccine	Pathogen target	Efficacy in pregnancy	Safety in pregnancy	Key recommendations	Common side effects
Bacterial Vaccines							
Tetanus Toxoid (TT)	Toxoid vaccine	Purified inactivated tetanus toxoid (from *Clostridium tetani*), aluminum adjuvant	*Clostridium tetani*	Prevents maternal and neonatal tetanus (88% reduction in neonatal deaths, 2000–2021).	No increased risk of adverse pregnancy outcomes.	WHO:5-dose series (early pregnancy, 6-8 wks later, 6-12 months after 2nd, 5 yrs after 3rd, 10 yrs after 4th) for unvaccinated pregnant women; China: 5-dose regimen (early pregnancy, ≥4 wks after 1st [≥2 wks pre-delivery], ≥6 months after 2nd, 1 yr later/next pre-pregnancy) for incomplete/unknown history; US CDC: 3-dose Td series (0, 4 wks, 6-12 months) for unvaccinated pregnant women.	Local injection site pain, headache,muscle pain,fever
Tdap (Tetanus-Diphtheria-Acellular Pertussis)	Combined inactivated/toxoid vaccine	Tetanus toxoid, diphtheria toxoid, acellular pertussis antigens	*Bordetella pertussis, C. tetani, Corynebacterium diphtheriae*	Reduces infant pertussis incidence in first 2 months of life.	No increased risk of chorioamnionitis, preterm birth, or adverse neonatal outcomes (3).	US CDC: Administer at 27–36 weeks in every pregnancy; UK/Australia: 20–32 weeks in every pregnancy.	Local injection site pain, fever, fatigue
Streptococcus Pneumococcal Vaccine*(e.g., PCV13, PPV23)*	Polysaccharide conjugate vaccine (PCV13) / Polysaccharide vaccine (PPV23)	PCV13: Purified capsular polysaccharides of 13 *Streptococcus pneumoniae* serotypes (conjugated to carrier protein); PPV23: Purified capsular polysaccharides of 23 *S. pneumoniae serotypes*	*Streptococcus pneumoniae*	Insufficient evidence for neonatal protection.	Not evaluated adequately in pregnant women.	No national guidelines recommend routine use in pregnancy (China, US, Australia).	Local injection site pain, fever, fatigue, headache,diarrhea,rash
Meningococcal Vaccine (MenACWY/MenB)	MenACWY: Conjugate subunit vaccine; MenB: Recombinant protein vaccine / Outer Membrane Vesicle (OMV) vaccine	MenACWY: Purified capsular polysaccharides of *Neisseria meningitidis* serotypes A/C/W/Y (conjugated to tetanus toxoid, diphtheria toxoid, or CRM197); MenB: ① Recombinant type (e.g., Bexsero): fHbp, NHBA, NadA, MenB OMV; ② Recombinant type (e.g., Trumenba): 2 variants of fHbp	*Neisseria meningitidis*	Reduces infant mortality in high-risk settings.	No association with adverse outcomes (6), but limited data.	US CDC: Permitted if risk > benefit; China: Contraindicated for use during pregnancy, especially in the first trimester.	Local injection site pain, fever, fatigue, headache,nausea,joint pain,muscle pain,chills
Group B Streptococcus (GBS) Vaccine	Conjugate subunit vaccine/Capsular polysaccharide vaccine	Not yet marketed; potential component adjustments	*Streptococcus agalactiae*	Induces maternal IgG transfer to neonates (phase 2 data).	Similar safety to placebo in mother-infant pairs (10).	Investigational; no routine recommendation (efficacy/safety under evaluation).	Local injection site pain, fever, fatigue, headache,muscle pain,joint pain, diarrhea,nausea
Viral Vaccines							
Influenza Vaccine (*e.g.*, IIV4/RIV4)	Inactivated influenza vaccine (IIV4) / Recombinant influenza vaccine (RIV4)	IIV4: Inactivated influenza virus strains (2 A subtypes: H1N1, H3N2; 2 B lineages: Victoria + Yamagata), aluminum adjuvant. RIV4: Recombinant influenza HA protein (2 A subtypes: H1N1, H3N2; 2 B lineages: Victoria + Yamagata).	Influenza virus	Reduces maternal influenza hospitalization (14); protects infants <6 months.	No increased risk of preterm birth, low birth weight, or birth defects (18).	WHO/US CDC/China: Routine annual vaccination at any pregnancy stage; avoid LAIV4.	Local injection site pain, fever, fatigue, headache,joint pain, diarrhea,nausea,pruritus,pharyngalgia,bellyache,cough
COVID-19 Vaccine	mRNA/Viral Vector(Adenovirus) /Protein Subunit/Inactivated Virus	mRNA encoding SARS-CoV-2 spike protein/Recombinant adenovirus vector carrying SARS-CoV-2 spike protein gene/Purified SARS-CoV-2 spike protein (or subunits)/Inactivated whole SARS-CoV-2 virus particles	SARS-CoV-2	For details, see **Table 2**.	No increased risk of preterm birth, congenital malformations, or infant mortality (38–43).	WHO/US CDC/ACOG: Routine; China: Recommended for high-risk pregnancies.	Injection site pain,fatigue,headache,muscle pain,chills,fever,nausea,joint pain,injection site swelling,carditis(For details, see **Table 2**).
Respiratory syncytial virus vaccine(e.g., ABRYSVO)	Bivalent recombinant prefusion F protein subunit vaccine	Bivalent recombinant RSV prefusion F (prefusion F) protein (targets RSV subtypes A and B); no adjuvant	Respiratory Syncytial Virus	Prevents severe RSV-associated illness in infants (46).	Initial concerns: Potential association with elevated preterm birth risk (47); Post-marketing cohort: No increased preterm birth risk (48) .	US CDC: Single dose at 32–36 weeks’ gestation; UK JCVI: Offer from 28 weeks’ gestation.	Local injection site pain, fever, fatigue, headache,muscle pain,joint pain,diarrhea,nausea
Hepatitis B Vaccine(e.g., recombinant HBsAg vaccine)	Recombinant protein vaccine	Recombinant hepatitis B surface antigen (HBsAg) protein (expressed via *Saccharomyces cerevisiae, Pichia pastoris*, or CHO cells), aluminum adjuvant	Hepatitis B virus (HBV)	Prevents mother-to-child transmission (no fetal infection risk).	Safe (recombinant, non-live vaccine).	US CDC: Administer if pregnant women are HBsAg-negative and at risk; China: Prefer preconception vaccination; complete series if initiated during pregnancy.	Local injection site pain, fever, fatigue, diarrhea,nausea,headache,muscle pain
Hepatitis A Vaccine (HepA-I / HepA-L)	HepA-I: Inactivated vaccine; HepA-L: Attenuated live vaccine	HepA-I: Inactivated hepatitis A virus (HAV) strains; HepA-L: Attenuated live HAV strains	Hepatitis A virus	Prevents acute HAV infection in pregnant women, thereby reducing risk of preterm birth and pregnancy complications.	- HepA-I: 25-year study showed no vaccine-related birth defects (51); - HepA-L: China lists pregnant women as contraindicated; women of childbearing age need contraception for ≥3 months post-HepA-L.	US CDC: Vaccinate pregnant women at risk of HAV infection; China: Contraindicated for pregnant women; - Australia: Not recommended for pregnant/lactating women.	Local injection site pain, fever, fatigue, headache,diarrhea,nausea,upper respiratory tract infection,cough,rash,tympanitis
Polio Vaccine (OPV / IPV)	OPV: Oral attenuated live vaccine; IPV: Inactivated viral vaccine	OPV: Attenuated live poliovirus strains; IPV: Formaldehyde-inactivated poliovirus strains	Poliovirus	Prevents poliovirus infection, avoiding severe outcomes (e.g., lifelong paralysis) in pregnant women and indirect risks to fetus.	OPV: Exposed group has higher preterm birth rate; no differences in stillbirth, low birth weight, congenital anomalies, or mortality (52); IPV: Safer than OPV.	US CDC: Administer IPV to high-risk pregnant women (needing immediate protection) per adult schedule; China: IPV only for absolute necessity; OPV avoided in pregnancy; Australia: Not recommended for pregnant/breastfeeding women.	Local injection site pain,fever,diarhea,vomiting,rash
HPV Vaccine(Bivalent/Quadrivalent/Nine-valent)	Recombinant protein vaccine	Bivalent: HPV 16/18 L1 capsid protein; Quadrivalent: HPV 6/11/16/18 L1 capsid protein; Nine-valent: HPV 6/11/16/18/31/33/45/52/58 L1 capsid protein; aluminum adjuvant	Human papillomavirus	No data on maternal/fetal protection during pregnancy.	Potential increased risk of spontaneous abortion with 9-valent vaccine (55) (limited data).	China/US CDC/Australia: Not recommended; delay until after lactation.	Local injection site pain, fever, fatigue, headache,muscle pain,diarrhea,nausea,pruritus,pruritus

Td, Tetanus-diphtheria; fHbp, Factor H Binding Protein; NadA, Neisserial Adhesin A; NHBA, Neisserial Heparin Binding Antigen; OMV, Outer Membrane Vesicles; HA, Hemagglutinin; CHO, Chinese Hamster Ovary; JCVI, Joint Committee on Vaccination and Immunization.

## Vaccines for preventing bacterial infections

### Tetanus vaccine

Tetanus, an acute and specific infectious disease, is caused by the infiltration of *Clostridium tetani* into the human body through wound contamination. This condition predominantly affects individuals with traumatic injuries or burns, neonates born under unhygienic delivery conditions, and patients exposed to inadequately sterilized surgical instruments. Although tetanus can affect individuals of any age or demographic, its incidence and clinical severity are notably higher among unvaccinated neonates and pregnant women.

Maternal immunization with tetanus toxoid (TT) vaccine, an inactivated toxoid vaccine, has been demonstrated as an effective intervention for preventing both maternal and neonatal tetanus (MNT). The main component of the TT vaccine is detoxified tetanus toxin (toxoid), derived from the culture supernatant of *Clostridium tetani*. This toxoid retains immunogenicity, inducing the production of specific antibodies in the body, while losing toxic activity to avoid causing tetanus symptoms. In 1989, the 42nd World Health Assembly (WHA) issued a global call to eliminate MNT, defined as an incidence of fewer than one case per 1,000 live births in all regions. The integration of TT vaccination during pregnancy with improved obstetric hygiene practices has significantly reduced MNT-related mortality. As of 2024, only 10 countries remain noncompliant with the MNT elimination (MNTE) target.

According to World Health Organization (WHO) estimates, neonatal tetanus deaths declined by 88% between 2000 (200,000 deaths) and 2021 (24,000 deaths). To mitigate the risk of neonatal tetanus resulting from unsterile deliveries or other exposures, the WHO recommends that women of reproductive age receive TT immunization. This strategy confers passive immunity to infants during the vulnerable period from birth to 3 months of age.

The WHO immunization protocol specifies that unvaccinated pregnant women or women of childbearing age should receive a five-dose TT vaccination series, initiated as early as possible during pregnancy. In alignment with these guidelines, China’s public health policy mandates that pregnant women with incomplete or undocumented immunization records complete the five-dose regimen, prioritizing early antenatal administration. Conversely, the US Centers for Disease Control and Prevention (CDC) advises a three-dose series of tetanus-diphtheria (Td) vaccine for unvaccinated pregnant women.

### Pertussis vaccine

Pertussis, caused by *Bordetella pertussis*, is a highly contagious acute respiratory infection associated with substantial morbidity and mortality in infants. Globally, the WHO estimates approximately 24 million cases and 160,700 deaths annually among children under 5 years of age. The CDC reports that the majority of pertussis deaths occur in infants under 3 months of age. In the USA, pertussis causes approximately 20 pediatric deaths annually, whereas in the Netherlands, 150–180 children under 2 years of age require hospitalization each year, with one infant death reported annually (1). Pertussis is a vaccine-preventable disease, and maternal immunization has been established as an effective strategy to protect infants who are too young to receive primary vaccination. The tetanus, diphtheria, and acellular pertussis (Tdap) vaccine—a combined inactivated/toxoid vaccine integrating three antigen components—when administered during pregnancy, provides passive immunity to neonates, significantly reducing pertussis incidence in the first 2 months of life (2). Its main components include the following: (1) Tetanus component; (2) Diphtheria component: detoxified diphtheria toxin (toxoid) from *Corynebacterium diphtheriae*; (3) Acellular pertussis component: purified acellular antigens of *Bordetella pertussis*. Research shows that Tdap vaccination during pregnancy can significantly reduce the incidence of pertussis in newborns (2). The Tdap vaccine demonstrates a favorable safety profile in pregnant women. A large-scale cohort study involving 118,211 pregnant women, of whom 103,258 (87%) received Tdap during gestation, found no increased risk of chorioamnionitis, preterm birth, or adverse neonatal outcomes (3). The WHO recommends maternal pertussis vaccination as a supplementary strategy in regions with high infant pertussis incidence or mortality. Similarly, the CDC advises Tdap administration during every pregnancy, regardless of prior vaccination history, with the optimal timing at 27–36 weeks of gestation to maximize transplacental antibody transfer. Nonetheless, vaccination is considered beneficial at any stage of pregnancy. Several high-income countries have successfully integrated maternal pertussis immunization into national programs: The USA introduced Tdap vaccination for pregnant women in 2011 to provide before infant immunization. The UK incorporated pertussis vaccination for pregnant women into its national program in 2012. Australia includes maternal pertussis vaccination in its National Immunization Program (NIP), recommending administration between 20 and 32 weeks of each pregnancy, although vaccination can still be provided until delivery. These policies have contributed to a measurable decline in neonatal pertussis morbidity and mortality, highlighting the critical role of maternal immunization as a public health intervention.

### Streptococcus pneumoniae *vaccine*

*Streptococcus pneumoniae* (pneumococcus) is a leading invasive bacterial pathogen, contributing significantly to global morbidity and mortality, particularly among immunocompromised populations. Globally, pneumococcal diseases account for an estimated one million pediatric deaths annually. In the USA, pneumococcal pneumonia results in approximately 150,000 hospitalizations each year, with invasive pneumococcal disease (IPD)—including meningitis and bacteremia—causing 3,250 fatalities in 2019. No national guidelines currently endorse maternal pneumococcal vaccination due to insufficient evidence of neonatal protection. China has not included pregnant women in the target population for pneumococcal vaccines. The CDC recommends pneumococcal vaccination for all children under 5 years of age, adults over 65 years of age, and individuals aged 5–65 years with identified risk factors (4). Currently, the clinically most commonly used pneumococcal vaccines mainly include two types of subunit vaccines: (1) Pneumococcal polysaccharide vaccine (PPV, e.g., PPV23)—a polysaccharide-based subunit vaccine whose main component is purified capsular polysaccharides extracted from the cell wall of *Streptococcus pneumoniae* (typically covering 23 common pathogenic serotypes of *Streptococcus pneumoniae*); (2) Pneumococcal conjugate vaccine (PCV, e.g., PCV13, PCV15)—a conjugate subunit vaccine whose main component is *Streptococcus pneumoniae* capsular polysaccharides covalently bound to carrier proteins. However, there is currently no recommendation for pregnant women to receive the pneumococcal vaccine. The Australian Department of Health and Elderly Care (DOHAC) does not recommend that pregnant women receive pneumococcal vaccines.

### Meningococcal vaccine

The mortality rate of invasive meningococcal disease is very high, with a mortality rate of up to one-sixth according to WHO data. One-fifth of survivors may experience long-term sequelae such as hearing loss, seizures, limb weakness, and visual impairment. In 2020, the WHO proposed the vision of “towards a world free of meningitis” by 2030, which includes three goals: elimination of bacterial meningitis epidemics, reduction of cases of vaccine-preventable bacterial meningitis by 50% and deaths by 70%, reduction of disability, and improvement of quality of life after meningitis due to any cause. Meningococcus, Streptococcus pneumoniae, Haemophilus influenzae, and Group B *Streptococcus* are common pathogenic bacteria that cause bacterial meningitis. The main serotypes of meningococcal bacteria that cause meningitis are A, B, C, Y, and W135 groups. Research shows that receiving the meningococcal vaccine during pregnancy can reduce infant mortality rates (5). Although current research shows that use of meningococcal vaccines during pregnancy is not associated with adverse pregnancy outcomes (6). However, due to the lack of reproductive toxicity testing or clinical studies in experimental animals and pregnant women, China has classified pregnant women as a prohibited population for the ACYW135 meningococcal polysaccharide vaccine (a polysaccharide subunit vaccine, whose main component is purified capsular polysaccharides extracted from A, C, Y, and W135 serogroups of Neisseria meningitidis), especially during the first three months of pregnancy. There are currently quadrivalent (serotypes A, C, W, and Y) meningococcal conjugate vaccines (MenACWY) and serotype B meningococcal vaccines (MenB) available in the United States. MenACWY is a conjugate subunit vaccine, with its main component being capsular polysaccharides from A, C, W, and Y serogroups of Neisseria meningitidis covalently bound to carrier proteins; MenB includes two types: recombinant protein vaccine or outer membrane vesicle (OMV) vaccine. Due to limited data on MenB vaccination during pregnancy, CDC recommends delaying MenB vaccination be deferred in pregnant and lactating women unless the woman is at increased risk, and, after consultation with her healthcare provider, the benefits of vaccination are considered to outweigh the potential risks. If indicated, pregnancy should not preclude vaccination with MenACWY. The DOHAC in Australia does not recommend pregnant or lactating women receive the meningococcal vaccine.

### Group B Streptococcus vaccine

Group B *Streptococcus* (GBS) is an opportunistic pathogen that can transform from a colonized state to a pathogenic bacterium under certain conditions, leading to invasive GBS disease in pregnant women or newborns. For GBS colonization in pregnant women, without intervention, 50% will vertically transmit the bacterium to the fetus or newborn. Without antibiotic prophylaxis during childbirth, 1%–2% of these newborns will develop early-onset GBS disease (GBS-EOD), which can cause neonatal bacteremia, pneumonia, and meningitis (7). Prophylactic use of antibiotics during the perinatal period can effectively reduce the incidence of invasive GBS disease in neonates (8). The use of antibiotics during delivery in pregnant women carrying GBS can prevent early-onset invasive infections in newborns (within the first 7 days of birth) but does not protect against late-onset GBS disease (GBS-LOD) occurring between 7 days and 3 months after birth. Effective GBS vaccination has the potential to reduce the incidence of illness in mothers, fetuses, and infants. Currently, no GBS vaccines are widely marketed, and most candidates remain in clinical trials. The primary vaccine types entering clinical development are GBS capsular conjugate vaccines and capsular polysaccharide vaccines. Maternal vaccination with the Group B *Streptococcus* capsular conjugate vaccine can increase the serum IgG concentration against capsular polysaccharides in newborns at birth, thereby reducing the risk of Group B *Streptococcus* disease (9). Results of a phase 2 study in pregnant women indicate that the candidate vaccine GBS6 generally has good tolerability, produces strong maternal antibody responses, and can be effectively delivered to infants. The safety of the vaccine and placebo groups is similar in both mothers and infants (10). Further research is needed to determine the safety and efficacy of using the Group B *Streptococcus* capsular conjugate vaccine in pregnant women.

## Vaccines for preventing viral infections

### Influenza vaccine

Due to physiological changes in the immune, circulatory, and respiratory systems during pregnancy—including increased heart rate, higher oxygen consumption, and decreased lung capacity—pregnant women face a significantly higher risk of influenza infection (11). The probability of pregnant women becoming severely ill and requiring hospitalization after influenza infection is relatively high (12). In addition, influenza infection during pregnancy is associated with a decrease in the average birth weight of infants (13). Vaccination against influenza during pregnancy can reduce the risk of hospitalization for influenza by an average of 40% (14). At the same time, antibodies generated by maternal influenza vaccination can be transmitted to developing infants (15), providing protection against influenza and influenza-related hospitalization during the first few months of life (16), when infants are too young to receive vaccinations. Additionally, receiving the influenza vaccine during pregnancy can reduce the incidence of low birth weight and significantly lower the rate of premature birth (17). Influenza vaccines have a favorable safety profile, with multiple studies reporting no adverse effects on fetuses—including preterm birth, low birth weight, small-for-gestational-age infants, or major birth defects—following maternal vaccination (18). The WHO and Public Health England (PHE) recommend annual influenza vaccination for all pregnant women. The Advisory Committee on Immunization Practices (ACIP) recommends that all individuals over 6 months of age, including pregnant women, receive the influenza vaccine. The CDC recommends that inactivated influenza vaccine (IIV4) or recombinant influenza vaccine (RIV4) can be administered at any time during pregnancy (before or during the flu pandemic), and recommends that September and October are usually good months to receive the flu vaccine. This vaccination can provide immunity to infants born during the upcoming flu season. However, it is not recommended for pregnant women to receive the live attenuated influenza vaccine (LAIV4). The Chinese National Immunization Program Technical Working Group recommends that pregnant women may receive influenza vaccines at any stage of pregnancy. Similarly, the DOHAC in Australia recommends influenza vaccination for pregnant women. Furthermore, Australia has included influenza vaccination during pregnancy in its NIP.

### COVID-19 vaccine

Current research has found that pregnant women infected with severe acute respiratory syndrome coronavirus-2 (SARS-CoV-2) have higher rates of ICU admission, need for invasive ventilation, need for extracorporeal membrane oxygenation (ECMO), and mortality compared to nonpregnant women (19, 20). The main pregnancy-related complications reported in pregnant women infected with SARS-CoV-2 include premature birth, stillbirth, preeclampsia, intrauterine growth restriction, and a higher risk of neonatal developmental defects (21). Pregnant women can obtain IgG antibodies by receiving the COVID-19 vaccine during pregnancy; these antibodies can be detected in the umbilical cord blood at delivery (22), providing protection to infants against COVID-19 infection (23). Currently, four main types of COVID-19 vaccines are marketed globally (23–32), with detailed information presented in **Table 2**. A cohort study confirmed that pregnant and lactating women exhibit robust vaccine-induced immune responses, with SARS-CoV-2 neutralizing antibody titers comparable to those in nonpregnant adults. These antibodies are efficiently transferred to fetuses *via* the placenta and to infants through breast milk (33). Regarding protective efficacy for infants, one study found that completing two doses of the messenger RNA (mRNA) COVID-19 vaccine during pregnancy may reduce the risk of COVID-19-related hospitalization in infants under 6 months, with an effectiveness rate of 61% (95% confidence interval [CI] = 31%–78%) (34). Another study found that administering two doses of the COVID-19 vaccine to pregnant women during pregnancy was 95% effective against SARS-CoV-2 Delta variant infection, and 97% effective in preventing related hospitalization in infants under 6 months (35). For maternal infection prevention, vaccination with the BNT162b2 mRNA vaccine was associated with a 78% reduced risk of SARS-CoV-2 infection in a 1:1 matched retrospective cohort (7,530 vaccinated vs. 7,530 unvaccinated pregnant women), confirming the efficacy of maternal vaccination against SARS-CoV-2 (36). Overall, receiving the COVID-19 vaccine during pregnancy can reduce the incidence of SARS-CoV-2 infections, including severe cases, lower the risk of COVID-19-related hospitalizations maternal mortality, and decrease the risk of SARS-CoV-2 infection in infants under 6 months (37). In terms of safety, evidence indicates that COVID-19 vaccination during pregnancy is safe and does not increase the risk of premature birth, low body weight, congenital malformations, or infant mortality (38–40). Multiple high-quality studies provide detailed supporting data. For example, a surveillance study included 35,691 pregnant participants, of whom 3,958 were enrolled in a pregnancy registry and received mRNA COVID-19 vaccines. Among these 3,958 participants, 827 had completed pregnancies, with a pregnancy loss rate of 13.9% and preterm birth rate of 9.4%—both comparable to prepandemic rates—and no vaccine-related severe adverse events were reported (41). An observational study of pregnant women who received the mRNA COVID-19 vaccine found that among 57 women who delivered during follow-up, there were no cases of preterm birth < 37 weeks or fetal/neonatal death (42). A study analyzing delivery outcomes of 2,002 pregnant women (140 vaccinated, 1,862 unvaccinated) found that the composite adverse outcome rate (5.0% vs. 4.9%) and preterm birth rate were not significantly different between the vaccinated and unvaccinated groups (43).

**Table 2:** Characteristics, efficacy and side effects of major currently used globally available covid-19 vaccines.

Vaccine name	Vaccine characteristics	Efficacy	Common side effects
Pfizer-BioNTech (BNT162b2)/Moderna (mRNA-1273)	mRNA	Against COVID-19-related hospitalization in infants: 35% efficacy (95% CI:15%-51%) for infants <6 months and 54% (95% CI:32%-68%) for infants <3 months (23).	- Injection site pain,fatigue,headache,muscle pain,chills,fever,nausea,joint pain,injection site swelling,carditis - No association with miscarriage, preterm birth, or congenital anomalies identified (30, 41).
Johnson& Johnson (Ad26.COV2.S)	Viral Vector (Adenovirus)	Against moderate to severe-critical COVID-19 (onset ≥14 days): efficacy 66.9%; onset ≥28 days: efficacy 66.1% (25) .	- Injection site pain,fatigue -some European countries restrict its use due to thromboembolic risk (24).
Novavax (Nuvaxovid)	Protein Subunit + AS03 Adjuvant	89.7% protection against SARS-CoV-2 infection (26).	-Injection site pain,chest pain,headache,fatigue,muscle pain,myocarditis and pericarditis (27).
Sinopharm (BBIBP-CorV)/Sinovac (CoronaVac)	Inactivated Virus	59.0%-90% protection against SARS-CoV-2 infection, 64.6%-95% against hospitalization, 92% against severe disease, and 79%-97% against death, 69.0% against ICU admission (29, 31).	- Injection site pain,fatigue,headache,fever,muscle pain,diarrhea,nausea,cough ,Bell's palsy (28, 32)

The WHO believes that the benefits of COVID-19 vaccination during pregnancy outweigh the potential risks and recommends that pregnant women receive the COVID-19 vaccine. The American College of Obstetricians and Gynecologists (ACOG) considers the COVID-19 vaccine safe for pregnant women and the fetus during any trimester. The COVID-19 vaccine generates antibodies that are passed to the fetus, which may protect against COVID-19 until a baby is eligible for vaccination at 6 months. The CDC recommends that people (including those who are pregnant, might become pregnant, were recently pregnant, or are breastfeeding) and infants 6 months of age or older be vaccinated with the latest version of the COVID-19 vaccine. The Society of Perinatal Medicine of the Chinese Medical Association suggests that, considering the inherent adverse effects of vaccination during pregnancy, COVID-19 vaccination may be postponed if there is no obvious risk of infection. However, if a pregnant woman faces a risk of infection—particularly during epidemics in daily life and/or workplace—it is recommended to receive an inactivated COVID-19 vaccine according to the standard procedure at any stage of pregnancy. Similarly, the DOHAC in Australia and the UK Medicines and Healthcare Products Regulatory Agency both recommend COVID-19 vaccination for pregnant women to help protect them and their infants from serious illness. The International Society of Infectious Diseases in Obstetrics and Gynecology (ISIDOG) recommends that pregnant women receive the COVID-19 vaccine and advises the use of mRNA-based vaccines (44).

### Respiratory syncytial virus vaccine

Respiratory syncytial virus (RSV) is a common respiratory virus and an important cause of morbidity and mortality in infants and young children worldwide. It is estimated that 2.0% of deaths in children aged 0 to 5 years and 3.6% of deaths in children aged 28 days to 6 months can be attributed to RSV (45). In 2019 alone, an estimated 33 million RSV-associated ALRTI cases occurred in children under five, resulting in 101,400 deaths worldwide (45). Given the persistent absence of efficacious antiviral therapeutics for RSV, prophylactic interventions constitute the cornerstone of disease management. Maternal RSV vaccination has emerged as a promising strategy to reduce severe RSV-related disease in infants. Clinical evidence demonstrates high protection against severe RSV-associated illness following vaccination during pregnancy, with efficacy rates of 81.8% within 90 days postpartum and 69.4% within 180 days (46). However, some studies suggest a potential association between maternal RSV vaccination and an elevated risk of preterm birth (47). The evolving landscape of RSV prophylaxis witnessed a paradigm shift in 2023 when the US Food and Drug Administration (FDA) granted approval to Pfizer’s bivalent RSV prefusion F protein subunit vaccine (ABRYSVO) for maternal immunization. Notably, while GSK’s adjuvanted recombinant vaccine (AREXVY)—an adjuvanted recombinant RSV protein subunit vaccine with main components of recombinant RSV prefusion F protein and AS01E adjuvant—and Moderna’s mRNA-1345 (mRESVIA)—an RSV mRNA vaccine with main components of mRNA encoding RSV prefusion F protein, lipid nanoparticles (LNP), and trace buffers—have received regulatory authorization for older adults (≥ 60 years), their safety profiles preclude antenatal application, establishing ABRYSVO as the sole maternal RSV vaccine globally. A cohort study involving 2,973 pregnant individuals post-marketing showed that among 1,026 participants (34.5%) who received prenatal RSVpreF vaccination, there was no association between prenatal RSVpreF vaccination and an increased risk of preterm birth (OR = 0.88; 95% CI: 0.64–1.20) (48). As of February 2025, the RSV immunoprophylaxis armamentarium encompasses five approved biologics categorized by target populations (**Table 3**). Accordingly, the CDC recommends a single dose of ABRYSVO at 32–36 weeks’ gestation. Similarly, the UK’s Joint Committee on Vaccination and Immunization (JCVI) advises offering RSV vaccination to pregnant individuals from 28 weeks’ gestation, with implementation scheduled to begin in September 2024.

**Table 3:** Approved respiratory syncytial virus (RSV) vaccines and monoclonal antibodies.

Manufacturer	Brand (generic)	Type	Target population	Use in pregnancy
GlaxoSmithKline	AREXVY	Protein subunit vaccine	Adults aged 50+	Contraindicated
Pfizer	ABRYSVO	Protein subunit vaccine	Adults aged 18+ and pregnant individuals (32–36 weeks gestation)	Approved
Moderna	mRESVIA (mRNA-1345)	mRNA vaccine	Adults aged 60+	Not currently approved
AstraZeneca/Sanofi	BEYFORTUS (Nirsevimab)	Monoclonal antibody	Prevention of RSV lower respiratory tract disease in Neonates and infants entering or during their first RSV season. Children up to 24 months of age who remain vulnerable to severe RSV disease through their second RSV season.	Not applicable
AstraZeneca	SYNAGIS (Palivizumab)	Monoclonal antibody	Children born at 35 weeks of gestation or less and less than 6 months of age at the onset of the RSV season. Children less than 2 years of age and requiring treatment for bronchopulmonary dysplasia within the last 6 months. Children less than 2 years of age and with haemodynamically significant congenital heart disease.	Not applicable

### Hepatitis B vaccine

Mother-to-child transmission is an important source of hepatitis B cases. The timing of transmission usually occurs during childbirth or the postpartum period. Hepatitis B virus (HBV) does not directly cause disease, and even if the fetus is exposed to the virus, fetal infection rarely occurs (49). However, during labor (including cesarean section), if the fetus or newborn is exposed to the mother’s blood and other bodily fluids, the virus can enter the newborn’s body. Close contact between newborns and their mothers after birth can also lead to transmission. Globally, the mainstream hepatitis B vaccine is a recombinant hepatitis B surface antigen (HBsAg) protein vaccine, with main components varying by expression system: (1) those produced *via Saccharomyces cerevisiae*—recombinant HBsAg protein and aluminum hydroxide adjuvant; (2) those produced *via Pichia pastoris*—recombinant HBsAg protein and aluminum adjuvant; and (3) those produced *via* Chinese hamster ovary (CHO) cells—recombinant HBsAg protein and aluminum phosphate adjuvant. As a recombinant protein vaccine, it contains no live hepatitis B virus and has no adverse effects on the fetus; therefore, pregnancy is not a contraindication for hepatitis B vaccine inoculation. However, the latest two hepatitis B vaccines—Heplisav-B and PreHevbrio—are not recommended for pregnant women due to insufficient data in this population. The CDC recommends that all pregnant women be screened for hepatitis B, and that adults aged 19–59 (including pregnant women) be vaccinated against hepatitis B. Newborns of HBsAg-positive pregnant women are at high risk of HBV infection. *China’s Clinical Guidelines for Prevention of Maternal to Infant Transmission of Hepatitis B Virus* (2020) recommend screening for hepatitis B serological indicators before pregnancy, and it is best to receive the hepatitis B vaccine prior to conception. However, if a woman becomes pregnant during the vaccination period, no special treatment is required, and the full vaccination course can be completed. According to the recommendations of the DOHAC in Australia, it is generally not recommended for pregnant or lactating women to receive the hepatitis B vaccine.

### Hepatitis A vaccine

Hepatitis A virus is an acute intestinal infectious disease caused by the hepatitis A virus (HAV), mainly characterized by damage to liver parenchymal cells. Acute HAV infection in pregnant women may increase the risk of preterm birth and pregnancy complications (50). Currently, inactivated hepatitis A vaccine (HepA-I) and attenuated live hepatitis A vaccine (HepA-L; main components: attenuated live HAV strains with reduced pathogenicity that can induce an immune response without causing severe disease) are available globally. A study summarizing safety data from pregnant women vaccinated with HepA-I showed that among 378 pregnant women who received HepA-I over a 25-year period, no birth defects related to vaccination were observed in pregnancy outcomes (51). The CDC recommends that pregnant women be vaccinated with the HepA vaccine if they are identified as being at risk for HAV infection during pregnancy. However, China has listed pregnant women as a contraindicated group for the hepatitis A vaccine and recommends that women of childbearing age use contraception for at least 3 months after receiving HepA-L injection. The DOHAC in Australia also does not recommend that pregnant or lactating women receive the hepatitis A vaccine.

### Polio vaccine

Poliovirus can cause poliomyelitis and lifelong paralysis. There are two types of polio vaccines: oral polio attenuated live vaccine (OPV) and inactivated polio vaccine (IPV), an inactivated viral vaccine with main components of formaldehyde-inactivated poliovirus strains (trivalent IPV [tIPV] containing serotypes 1, 2, 3 or monovalent IPV [mIPV] for single serotype) and an aluminum adjuvant in most formulations. Current evidence shows that exposure to OPV during pregnancy does not result in differences in stillbirth rates, low birth weight, congenital anomalies, neonatal mortality, or maternal mortality compared to nonexposed groups; however, the preterm birth rate is higher in the exposed group (18.4% vs. 11.0%, *p* = 0.011) (52). The CDC recommends that if a pregnant woman is at increased risk for infection and requires immediate protection against polio, IPV can be administered in accordance with the recommended schedules for adults. The instructions for the IPV launched in China state that pregnant women can only be vaccinated when it is absolutely necessary. The DOHAC in Australia does not recommend that pregnant or breastfeeding women receive the polio vaccine.

### HPV vaccine

Human papillomavirus (HPV) infection is typically transmitted through direct contact with infected organs (via skin or mucous membranes) and is the most common sexually transmitted infection. Multiple research reports indicate that HPV-positive women have an increased risk of adverse pregnancy outcomes, including premature birth, miscarriage, gestational hypertension, intrauterine growth restriction, low birth weight, premature rupture of membranes, and fetal death (53). Currently, there is no evidence to suggest that administering bivalent HPV vaccines (a recombinant HPV protein subunit vaccine; main components: recombinant L1 capsid proteins of HPV types 16 and 18, plus aluminum hydroxide adjuvant) during pregnancy increases the risk of teratogenicity (54). However, a systematic review and meta-analysis showed that receiving the bivalent HPV vaccine within 45 days before the last menstrual period appears to increase the risk of spontaneous abortion (1.59 [1.04–2.45]) (55). Another systematic review showed that unintentional vaccination with bivalent HPV during pregnancy did not significantly increase the risk of miscarriage, stillbirth, small for gestational age, premature birth, or birth defects (56). Exposure to the quadrivalent HPV vaccine (a recombinant HPV protein subunit vaccine; main components: recombinant L1 capsid proteins of HPV types 6, 11, 16, and 18, plus aluminum adjuvant) during pregnancy or the peri-pregnancy is not associated with adverse pregnancy or delivery outcomes (57). A retrospective study in China found no stillbirths among 50 pregnant women who received the nine-valent HPV vaccine (a recombinant HPV protein subunit vaccine, main components: recombinant L1 capsid proteins of HPV types 6, 11, 16, 18, 31, 33, 45, 52, and 58, plus aluminum adjuvant), but one case of microtia was observed (58). A systematic review and meta-analysis showed that pregnancy exposure to the nine-valent HPV within 30 days of conception appeared to increase the risk of spontaneous abortion (2.04 [1.28–3.24]) (55). Due to limited data on HPV vaccination during pregnancy, China does not recommend prophylactic HPV vaccination for pregnant women. Women planning pregnancy in the near future are advised to postpone vaccination until after lactation. If an unexpected pregnancy occurs after initiating vaccination, incomplete vaccine doses should be discontinued. If the vaccination series has been completed, no intervention is required. Similarly, the CDC does not recommend HPV vaccination during pregnancy and advises that if a woman is found to be pregnant at the start of the vaccination schedule, the remaining doses should be postponed until after pregnancy. No pregnancy test is required before vaccination. If a vaccine dose is inadvertently administered during pregnancy, no intervention is needed. The DOHAC in Australia does not recommend HPV vaccination for pregnant women; however, lactating women may receive the vaccine.

## Discussion

Pregnancy represents a unique immunological state characterized by maternal immune modulation, which increases susceptibility to viral and bacterial pathogens, thereby elevating risks of adverse outcomes, including severe infections, preterm birth, miscarriage, and stillbirth. Neonates remain vulnerable during the first months of life due to immature humoral immunity and incomplete vaccine-induced protection. Current evidence indicates that administration of inactivated viral/bacterial vaccines and toxoids during pregnancy does not pose fetal risks (59), with most nationally recommended maternal vaccines falling into this category. For instance, the US CDC, ACOG, and UK Health Security Agency endorse prenatal vaccination against influenza, pertussis, COVID-19, RSV, hepatitis B, and tetanus.

Live-attenuated vaccines, including measles, mumps, and rubella (MMR), varicella, and oral poliovirus vaccines, are generally contraindicated during pregnancy due to theoretical fetal risks, with postvaccination contraception advised for 28 days post-MMR/varicella administration. While unintended exposures require fetal risk counseling, termination is not indicated. Vaccines such as pneumococcal, meningococcal, and HPV require further safety validation, though meningococcal and hepatitis A vaccines may be administered under risk–benefit analysis.

Despite established safety profiles for pertussis and influenza vaccines, global coverage remains suboptimal (60, 61), attributed to sociopsychological barriers such as vaccine hesitancy, perceived low infection risk, and misinformation (62). Disparities in maternal immunization rates underscore the need for robust clinical evidence to address knowledge gaps and mitigate misconceptions. Multifaceted interventions targeting healthcare provider education, public health messaging, and equitable access are critical to optimizing prenatal vaccine uptake and safeguarding maternal–neonatal health outcomes.
